# CS-6-induced p62 accumulation exacerbates DNA damage in colorectal cancer

**DOI:** 10.3389/fphar.2025.1568339

**Published:** 2025-05-09

**Authors:** Yitong Gong, Menghan Dong, Xiaofei Feng, Naiyu Zhang, Xuehui Cui, Liushuyue Wang, Qi Qi, Chiu-fai Kuok, Qingling Jiang, Sixue Bi

**Affiliations:** ^1^ Featured Laboratory for Biosynthesis and Target Discovery of Active Components of Traditional Chinese Medicine, School of Traditional Chinese Medicine, Binzhou Medical University, Yantai, Shandong, China; ^2^ School of Humanities, Ludong University, Yantai, Shandong, China; ^3^ Yantai Affiliated Hospital of Binzhou Medical University, The Second Clinical Medical College of Binzhou Medical University, Yantai, Shandong, China; ^4^ Faculty of Health Sciences and Sports, Macao Polytechnic University, Macao, SAR China

**Keywords:** gamabufotalin (CS-6), colorectal cancer (CRC), DNA damage, p62, autophagy

## Abstract

**Background:**

Gamabufotalin (CS-6), a bufadienolide derived from Chansu, has been reported to exhibit anti-tumor effects in various cancers, including glioblastoma, nonsmall cell lung cancer, and breast cancer. However,its role in colorectal cancer (CRC) remains unexplored.

**Objective:**

Our study aimed to evaluate the inhibition of CS-6 to CRC cells by cell viability assay, colony formation assay, comet assay, and cell cycle analysis firstly. And its molecular mechanism was studied by immunofluorescence (IF) assay, western blot (WB) assay, siRNA transfection, protein-protein interaction (PPI) network and co-immunoprecipitation (Co-IP) assay. Finally, the in vivo antitumor assessments of CS-6 on colorectal cancer was validated through an transplant colorectal cancer model.

**Results:**

CS-6 treatment significantly inhibited CRC SW620 and DLD1 cell viability and colony formation *in vitro*. Furthermore, CS-6 treatment-induced DNA damage and cell cycle arrest in SW620 and DLD1 cells. The western blot assay revealed that CS-6 treatment upregulated p62 expression. Knockdown of p62 in this study significantly alleviated CS-6-induced DNA damage and the downregulation of cyclin expression in SW620 and DLD1 cells. Additionally, the results indicated increased expression of microtubuleassociated protein I/II light chain 3II (LC3II) and reduced binding between B-cell lymphoma-2 (Bcl2) and beclin-1, suggesting that CS-6 treatment activated early-stage autophagy in CRC cells. However, inhibition of latestage autophagy and autophagy-related protein 5 (ATG5) with chloroquine and si-ATG5, respectively, further indicated that CS-6-induced autophagy defects led to p62 accumulation, exacerbated cell proliferation inhibition, and aggravated DNA damage. Intraperitoneal injection with CS-6 inhibited tumor growth in nude mice with colorectal cancer, and promoted the protein expression of phosphorylated H2A histone family member X (γH2AX), p62, phosphorylated Ataxia-telangiectasia mutated kinase (p-ATM) and LC3 I/II.

**Conclusion:**

This study suggests that CS-6 may exert its anti-tumor effects in CRC by inducing autophagy defects, resulting in p62 accumulation and DNA damage *in vitro* and *in vivo*.

## 1 Introduction

Colorectal cancer (CRC) ranks as the most prevalent gastrointestinal cancer, characterized by a high mortality rate and poor prognosis (Sung et al., 2021; Akimoto et al., 2021). Common treatments for CRC encompass surgery, radiation therapy, targeted therapy, and chemotherapy (Fan et al., 2021). In recent years, immunotherapies such as immune checkpoint inhibitors (e.g., PD-1/PD-L1 inhibitors) and adoptive cell therapies (e.g., CAR-T) have emerged as promising strategies, particularly for microsatellite instability-high (MSI-H) or mismatch repair-deficient (dMMR) CRC subtypes. These therapies enhance anti-tumor immune responses by overcoming immune evasion mechanisms, offering durable clinical benefits in select patient populations. Additionally, alternative therapies, including natural products and Traditional Chinese Medicine (TCM)-derived compounds, are being explored for their potential to synergize with conventional treatments while minimizing toxicity (Morazán-Fernández et al., 2022; Zhu et al., 2024). Despite recent advancements in treating primary and metastatic CRC, the projected prognosis and mortality rates for 2023 do not indicate significant improvements in cure rates or the long-term survival of CRC patients (Siegel et al., 2023). Furthermore, chemotherapy and radiotherapy medications can lead to various adverse side effects, including nervous system abnormalities (Godlewski and Kmiec, 2020), gastrointestinal reactions (Keum and Giovannucci, 2019), and myelosuppression (Yang et al., 2023), negatively impacting the quality of life of patients. An urgent requirement exists for the development of non-toxic, safe, and efficacious alternative drugs to treat colorectal cancer (CRC).

Long-lasting mutations occurring during DNA replication can cause DNA damage, influencing the genetic makeup of an individual (Nastasi et al., 2020). Additionally, the failure to promptly repair DNA damage can result in cell death (Ciechomska, 2018). Research has shown that uncontrolled mutations and disruptions in genomic stability may lead to tumor heterogeneity and polymorphism, potentially activating anti-tumor immune responses (Gillman et al., 2021). This includes the mutation of key genes within tumor cells, stalling of the replication fork, and the induction of autophagy and apoptosis (Sarmah et al., 2021). Autophagy and DNA damage repair are not two distinct processes in tumorigenesis, but rather perform many, overlapping, and complimentary activities. When damaged DNA cannot be repaired, overactive autophagy prevents tumor growth by inhibiting cell proliferation and death (Liu et al., 2023). Autophagy is a highly conserved physiological process in eukaryotic cells. It involves the engulfing of faulty proteins, damaged organelles, or other cytoplasmic components by a bilayer autophagosome, which subsequently combines with a lysosome to form an autophagolysosome, resulting in the degradation of its contents. However, excessive autophagy can lead to cell apoptosis and aging, ultimately inhibiting tumor formation (Liu et al., 2015). P62, a stress-responsive protein, exerts a regulatory role in several pathways linked to cancer development (Tao et al., 2020). Moreover, p62 serves as a key regulator in several cancer types, such as breast cancer (Qi et al., 2022) and liver cancer (Duran et al., 2016), playing a pivotal role in the pathogenesis of various diseases. Furthermore, p62 exhibits dynamic associations with various pathways in case of severe DNA damage, associating with several DNA damage repair pathways, including homologous recombination repair, non-homologous recombination repair, and DNA damage response (DDR) pathways, activate autophagy to prevent tumor growth (Filomeni et al., 2015; Lyu et al., 2021) DNA damage foci and regulates DNA repair (Li et al., 2022). Positioned as an adaptable protein between the autophagosome and its substrate, p62 is selectively wrapped into the autophagosome, acting as a bridge between microtubule-associated proteins I/II light chain 3II, commonly known as LC3, the protein that plays a significant role in cellular processes, particularly in autophagy and polyubiquitination proteins (Kageyama et al., 2021). Subsequently, it undergoes degradation by proteolytic enzymes within the autophagolysosome.

Animals, plants, and microorganisms serve as abundant sources of natural products (Naeem et al., 2022; Si et al., 2018; Chen et al., 2012). Naturally-derived compounds possess complex structures and a wide array of pharmacological activities, contributing significantly to the development of numerous chemotherapeutic drugs, such as vincristine (Lien et al., 2021) and taxol (Yang and Horwitz, 2017). Several animal-derived substances, including Cinobufagin (Xiong et al., 2019) and Lytta vesicatoria (Rauh et al., 2007), have exhibited potential anti-tumor properties. Among them, Gamabufotalin (CS-6) is a bufodienolactone comprising 1.76%–4.97% of toad venom, a traditional Chinese medicine derived from dried toad skin and parotid venom (Rauh et al., 2007; Asrorov et al., 2023). Studies have unveiled the efficacy of CS-6 against various malignant tumors, including glioblastoma (Yuan et al., 2019), non-small cell lung cancer (Hou et al., 1997), and breast cancer (Forde and Ettinger, 2013; Liu et al., 2019). Thus, the effects and underlying mechanisms of CS-6 on CRC are yet to be fully elucidated. There are currently studies reporting that the accumulation of p62 can cause autophagic flow blockage, thereby exacerbating DNA damage, but there are no reports on the mechanism of action of related drugs. Our research findings indicate that CS-6 concurrently induces DNA damage and p62 accumulation in CRC cells, thus triggering autophagy defect inducing cell death to mediate its anti-tumor activity.

## 2 Materials and methods

### 2.1 Cell culture and drug therapy

The human colorectal cancer (CRC) cell lines, SW620 (ECACC identifier: 87051203) and DLD1 (ECACC identifier: 90102540) were obtained from the European Collection of Authenticated Cell Cultures (ECACC). These cell lines were cultured in Dulbecco’s modified Eagle’s medium (DMEM) from Sigma-Aldrich, USA, supplemented with 10% fetal bovine serum (Gibco, USA) and 1% penicillin-streptomycin (Solarbio, Beijing, China). Cells were maintained in a humidified environment at 37°C with 5% CO_2_. The experimental compound, CS-6, was supplied by Shandong Luye Pharma LTD (Yantai, Shandong, China). CS-6 for experiments was prepared by dissolving it in dimethyl sulfoxide (DMSO) obtained from MedChemExpress, USA. In some experiments aimed at inhibiting autophagic flux, cells were treated with 10 µM chloroquine (CQ) sourced from MedChemExpress, USA, for a duration of 24 h.

### 2.2 Cell viability assay

To evaluate the short-term impact of CS-6 on cell growth, a Cell Counting Kit-8 (CCK-8) detection assay was executed (MedChemExpress). In this assay, cell suspensions were meticulously distributed into individual wells of a 96-well plate, with each well receiving 10 μL of the suspension. Subsequently, the plates were placed inside an incubator set to maintain a temperature of 37°C in an atmosphere containing 5% CO_2_. Following cell incubation, 5 µL of the CCK solution was added to each well (Asrorov et al., 2023), following standard protocols. The plates were once again incubated under the same conditions (37°C, 5% CO_2_) for a duration of 1–4 h. Finally, the absorbance at 450 nm was measured using an enzymatic marker, thereby enabling the assessment of cell growth in response to CS-6 treatment.

### 2.3 Colony formation assay (CFA)

SW620 and DLD1 cells were initially seeded in 6-well plates at a density of 500 cells per well and subjected to culturing for 24 h. Once adherent, the cells underwent exposure to various concentrations of CS-6 (5, 10, 20, 40, 80, 160, 320, and 640 nM) for an additional 24 h, while untreated cells served as the Control group. Following this 24-h treatment period, the cells were subjected to incubation for an additional 12 days, during which colonies formed from both the treated and untreated cells. The assessment of these colonies involved initial fixation using a 4% paraformaldehyde solution (Beyotime, Shanghai) for 15 (Teng et al., 2016), minutes, followed by washing with phosphate-buffered saline (PBS). Subsequently, the colonies were stained with a 0.1% crystal violet (Solarbio, Beijing, China) solution for 10 min. Lastly, the colonies were observed and photographed using an inverted microscope (Leica DMI3000B, Leica Microsystems CMS GmbH, Wetzlar, Germany), and the number of colonies with a diameter exceeding 0.5 mm was counted for further analysis. The number of colonies was manually counted using ImageJ.

### 2.4 Cell cycle analysis

The process involved in preparing the cells for analysis is as follows: Firstly, the digested cells (Thermo Fisher, trypsin; catalog number 25200056) were centrifuged at 900 rpm for 3 min. Thereafter, the cells were washed with phosphate-buffered saline (PBS) and centrifuged once more. They were then fixed by immersing them in pre-cooled 70% ethanol for a duration of 2 h at 4°C. Subsequently, another round of washing with PBS was carried out, followed by exposure to 100 µL of RNase A (catalog number CA1510; Solarbio). This treatment lasted for 30 min at 37°C, in accordance with the given instructions. Next, the cells underwent staining with 400 µL of propidium iodide (PI) staining solution for 30 min in a dark environment at 4°C.

Ultimately, the stained cells were analyzed using a fluorescence microscope, and the red fluorescence was captured at an excitation wavelength of 488 nm.

### 2.5 Immunofluorescence (IF) assay

Following CS-6 treatment, SW620 and DLD1 cells were cultured on 35-mm confocal dishes (BeyoGold™) and underwent a series of steps for immunofluorescence staining. The process of cell staining and preparation for microscopy involved several steps:

Initially, cells were subjected to a triple rinse with PBS to ensure the removal of any residual substances. Subsequently, they were fixed with 4% paraformaldehyde (Beyotime) for a duration of 15 min. Following fixation, three PBS washes, each lasting 3 min, were performed to eliminate excess paraformaldehyde.

To prevent nonspecific binding, the cells were blocked with bovine serum albumin (BSA) for 30 min at room temperature. Following this, the cells were subjected to an overnight incubation at 4°C with primary antibodies.

After the overnight incubation, the cells were subjected to three rounds of PBS washing, each lasting 3 min, with subsequent removal of any excess liquid. Following this, the cells were exposed to secondary antibodies for 1 h at room temperature on a shaker.

Another set of three PBS washes was conducted after the secondary antibody treatment. To visualize cell nuclei, the cells were counter-stained with DAPI for 15 min, followed by another PBS wash.

To minimize autofluorescence and enhance image quality, an anti-fluorescence quencher solution was applied. Finally, the stained cells were observed and photographed using a fluorescence microscope.

### 2.6 Comet assay

SW620 and DLD1 cells were cultured in dishes and subjected to treatment with CS-6 (80 nM) for 24 h. Afterward, the cells were exposed to pancreatic enzymes, rinsed with pre-cooled PBS in an ice bath, centrifuged to collect cell precipitates, and resuspended in PBS (1 × 10^6^ cells/mL). The first layer of gel was prepared on a glass slide using the Comet Electrophoresis Kit (KeyGEN BioTECH, Jiangsu, China) following the provided protocol. Subsequently, 10 µL of cell suspension was evenly mixed with 0.7% low melting point agarose, and 70 µL drops were swiftly added to the initial gel layer. These drops were covered with a cover glass and subjected to incubation at 4°C for 10 min. Next, the glass slide was placed in a 10-cm Petri dish, immersed in 10 mL pre-cooled lysate for 1–2 h at 4°C, and rinsed with PBS for 3 min. The slide was then positioned in a horizontal electrophoresis tank, filled with electrophoresis buffer up to 0.25 cm above the rubber surface of the slide, and subjected to electrophoresis at room temperature for 20–60 min under alkaline conditions to facilitate DNA uncoiling. Electrophoresis was conducted for 30 min at 25 V. The slide was placed onto a plate and subjected to 1–3 treatments with a neutral buffer at 4°C for 5–10 min each. Subsequently, the slides were exposed to 20 µL of PI solution for 10–20 min in a dark environment and rinsed three times with ultrapure water. Finally, the sides were covered with a cover glass and viewed under a fluorescence microscope. For each slide, 20 randomly chosennuclei were analysed using Komet v5.5 analysis software.

### 2.7 Western blot (WB) assay

The protein analysis process for SW620 and DLD1 cells followed the given instructions. Cells were lysed using a buffer containing protein phosphatase inhibitors (ThermoFisher, America). The protein content was quantified according to the instructions provided by a Bicinchoninic Acid Protein Assay Kit. In the next step, equal quantities of protein samples were subjected to separation through sodium dodecyl-sulfate (SDS) polyacrylamide gel electrophoresis, followed by their subsequent transfer onto polyvinylidene difluoride membranes. To block the membranes, exposure to 5% skim milk in 1× tris-buffered saline with Tween 20 (TBST) was done for over 2 h at room temperature. Following this process, the membranes were exposed overnight to primary antibodies in 1% BSA at 4°C. Afterward, after three TBST washes, the membranes were incubated with the appropriate secondary antibodies for 40 min at room temperature. Finally, the membranes were visualized using an automatic chemiluminescence image analysis system (Tanon-5200), and optical density measurements were performed using ImageJ v1.41 software (Bethesda, Maryland, United States). Anti-phosphorylated-ataxia telangiectasia mutated kinase (P-ATM), Anti-ataxia telangiectasia mutated kinase (ATM), Anti-LC3A/B and Anti-autophagy-related protein 5 (ATG 5) were purchased from Cell Signaling Technology (#13050, #2873, #12741, #12994, Boston, America). Anti-beclin-1 (BECN1), Anti-B-cell lymphoma-2 (BcL2), Anti-cyclin-dependent kinase 1 (CDK1) and Proteintec Anti-cyclin B1were prchased from Proteintech(11306-1-AP, 68103-1-Ig, 11306-1-AP, 68103-1-Ig, 19532-1-AP,28603-1-AP, Chicago, America). Anti-p62/SQSTM1and Anti-γH2AX were purchased from Abmart (T55546,T56572, Shanghai, China). Anti-β-actin was purchased from Solarbio (K101527P, Beijing, China).Anti-rabbit secondary antibodies and Anti-mouse secondary antibodies were purchased from Thermo Fisher (31460, 31430, Massachusetts, America).

### 2.8 siRNA transfection

SW620 and DLD1 cells were seeded in 6-well plates and incubated with p62 and ATG5 siRNAs (5′-GAGGAUCCGAGUGUGAAUUUCdTdT-3′ and 5′-AGG​UAC​UUU​CCU​CAA​UCA​CAT​T-3′, respectively (GenePharma, Shanghai, China) to suppress p62 and ATG5 expression. Following transfection with Lipofectamine 3000 (Invitrogen, USA), the cells were cultured in Opti-MEM reduced serum medium (31985070; ThermoFisher).Following exposed to 100 nM ATG5 and p62 siRNA for 8 h, the cells were incubated in a complete medium for 48 h and subsequently exposed to CS-6 treatment for 24 h. Finally, WB analysis and CCK-8 assays were carried out to evaluate protein expression levels and the viability of CRC cells.

### 2.9 Co-immunoprecipitation (Co-IP) assay

SW620 and DLD1 cells were treated with an immunoprecipitation lysate buffer (ThermoFisher, USA) containing protein phosphatase inhibitors (Solarbio, Beijing) and then centrifuged to obtain cell lysate. Protein A/G magnetic beads (MedChemExpress, USA) were combined with IgG antibodies (Beyotime, Shanghai) and BECN1 antibodies (ProteinTech, USA). The cell lysate was prepared following the specified instructions. Subsequently, the cell lysate was incubated with the antibody-magnetic bead complex at 4 °C overnight with gentle agitation. Afterward, the beads were washed thrice to eliminate any detergent residue, and they were then treated with 20 μL SDS buffer and boiled for 5 min. Finally, the bound proteins were determined by using the WB assay.

### 2.10 Proteome profiling and protein-protein interaction (PPI) network

Proteins were extracted from CS-6-treated SW620 cells for proteomic analysis (conducted by Shanghai Ouai Biotechnology Co. Ltd., Shanghai, China). Key genes and signaling pathways were identified via the protein interaction network of the differentially expressed genes (DEGs) using the GeNets database.

### 2.11 Immunohistochemistry (IHC)

The tumor tissue was fixed in 4% paraformaldehyde and then embedded in paraffin. The paraffin blocks were cut into 4.0 µm thick sections. After degreasing the slices in water, soak them in Tris EDTA buffer (pH 9.0), microwave on high heat for 3–4 min until boiling, and cool at room temperature. Hatch the slices in 3% H_2_O_2_ for 30 min to inhibit the activity of endogenous peroxidase. Washing the slices three times with distilled water. The slices was equilibrated to room temperature by 10% sheep serum, and histologically circled and sealed for 30 min. Remove residual serum in slices, repeat circular drawing with a chemical pen, and incubate sections overnight at 4 °C for primary antibody. The next day, the slices was washed three times, then incubated with secondary antibody at room temperature for 1 h, washed with PBST three times, each time for 5 min. Perform color reaction on slices using diaminobenzidine and peroxide (DBA-H_2_O_2_). Stain the slices with hematoxylin for 1–2.5 min and wash them three times with clean water. Sealing: Dehydrate the slices with ethanol gradient (from low to high: 50%, 70%, 95%, 100%) for 2 min each, xylene for 5 min, and then pack the slices. Evaluating the expression of γ H2A and p62 using ImageXpress Micro Confocal (Molecular Devices, LLC, Sunnyvale, CA, USA).

### 2.12 Haematoxylin-eosin (HE) staining

Liver and kidney tissues were collected and fixed in 4% paraformaldehyde at 4°C for>24 h. The tissues were dehydrated, individually embedded in wax blocks and cut into 4 μM sections. Next, the sections were baked, dewaxed, soaked in a gradient of ethanol, and washed with ultradistilled water. The sections were then stained with haematoxylin and eosin (HE) and sealed with neutral gel. Representative images were acquired under an inverted microscope (Leica DMI3000B, Leica Microsystems CMS GmbH, Wetzlar, Germany).

### 2.13 *In vivo* antitumor assessments of CS-6 on colorectal cancer

BALB/c-Nude mice (No. 202146794, ♀, 6 weeks old) were purchased from GemPharma Tech Biological Technology Co., Ltd. (Nanjing, Jiangsu, China). Animals were observed in quarantine for 7 days prior to use and raised in specific pathogen-free animal rooms. SW620 cells (5 × 10^7^) in 0.1 mL PBS were subcutaneously injected into the armpits of mice for tumorigenesis. The mice were randomly divided into two groups (8 mice/group) after tumor measurement. The mice in the two different groups did or did not receive 2.5 mg/kg CS-6 by intraperitoneal injection every other day. The tumor length (L) and width (W) were measured every 1 day by a Vernier calliper, and the tumor volume (Tv) was calculated according to the formula Tv = 1/2 × L × W^2^. After 14 days of treatment, the mice were sacrificed under anaesthesia. Meanwhile, the tumors and related organs were removed, weighed and used to carry out further experiments. All animal experiments were carried out in accordance with the ARRIVE guidelines and in accordance with the U.K. Animals (Scientific Procedures) Act, 1986 and associated guidelines. And our experiments were approved by the Ethics Committee for Animal Experiments of Binzhou Medical University (No. 2022-135).

### 2.14 Statistical analysis

Statistical analysis was conducted using GraphPad Prism 9.0 software. The data in this study were gathered from a minimum of three independent experiments. To compare two groups, a Student’s t-test was utilized, and for analyzing statistical differences among multiple groups, a one-way analysis of variance (ANOVA) was utilized. The statistical significance level was established at *P < 0.05.

## 3 Results

### 3.1 Inhibition of SW620 and DLD1 *c*ell proliferation by CS-6 *in vitro*

LOVO, HCT15, SW620 and DLD1 cells were subjected to varying concentrations of CS-6 (5, 10, 20, 40, 80, 160, 320, and 640 nM) for 24 h. Their cell viability and proliferative capacity were assessed using the CCK8 assay and colony formation assay (CFA).

**FIGURE 1:**
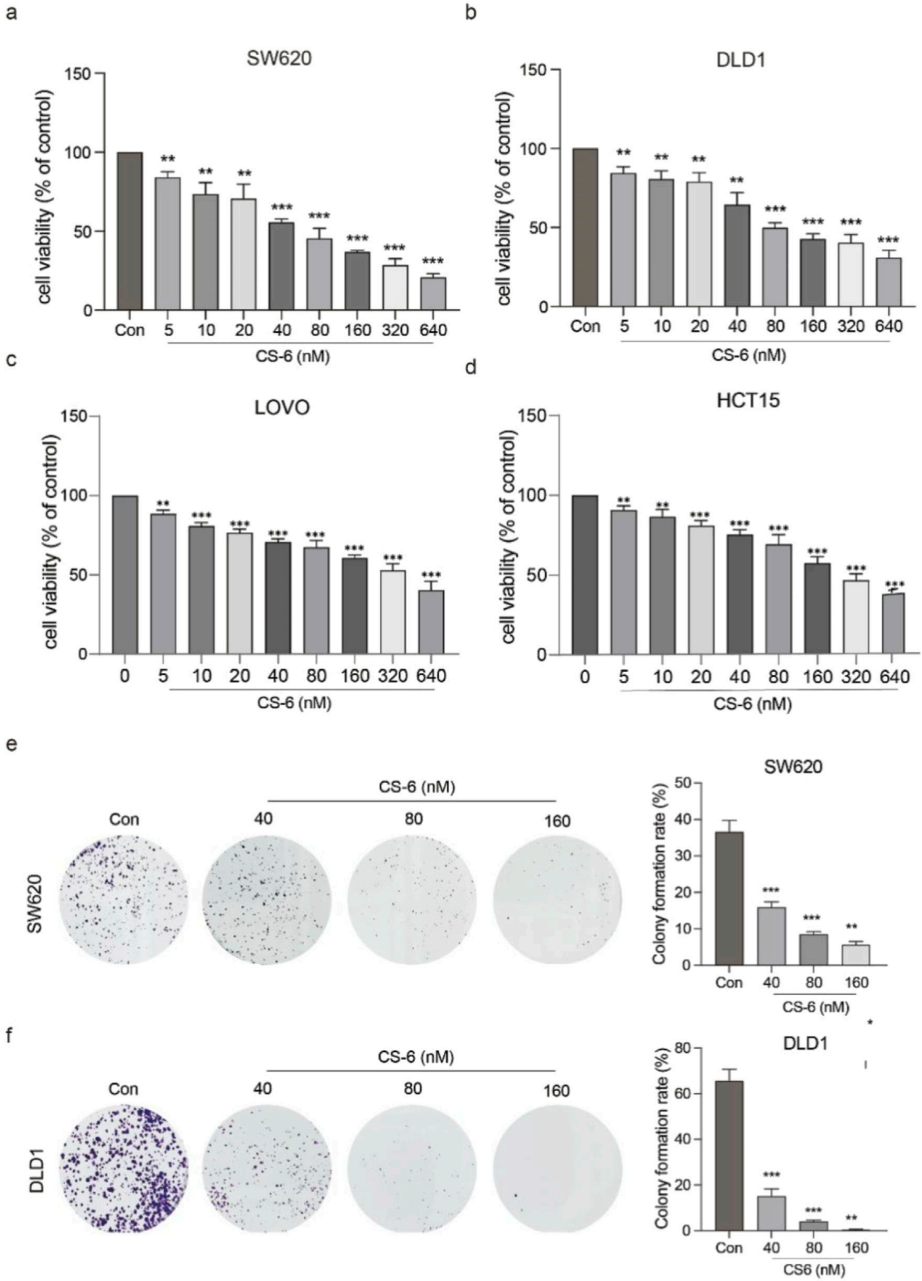
CS-6 inhibited the proliferation of coleractal and other cells *in vitro*. SW620, DLD1, HCT15 and LOVO cells were exposed to different concentrations of CS-6 (5, 10, 20, 40, 80, 160, 320, and 640 nM) for 24 h. The untreated cells served as the Control group. **(a–d)** Cell viability of both Control and CS-6-treated cells was assessed using the CCK8 assay. IC50 values of CS-6 for SW620, DLD1, HCT15 and LOVO cells are 65.58, 121.1, 313.4 and 245.65 nM respectively. **(e,f)** Additionally, the colony formation capability of Control and CS-6-treated SW620 and DLD1 cells was assessed using the colony formation assay. The presented values indicate the mean ± standard deviation obtained from three independent experiments. Significance levels are indicated as **p < 0.01 and ***p < 0.001.

The results from the CCK8 assay showed a concentration-dependent reduction in SW620, DLD1, HCT15 and LOVO cell viability, yielding IC50 values of 65.58 and 121.1 nM for SW620 and DLD1 cells, respectively (Figure 1a-d). Additionally, the CFA results demonstrated a significant decrease in the number of colonies formed by SW620 and DLD1 cells at concentrations of 80 nM and 160 nM (Figure 1e,f). These findings collectively highlight the inhibitory effect of CS-6 on the proliferation of CRC cells *in vitro*.

### 3.2 Induction of G2/M phase arrest and modulation of cell cycle-related proteins by CS-6 in SW620 and DLD1 cells

**FIGURE 2:**
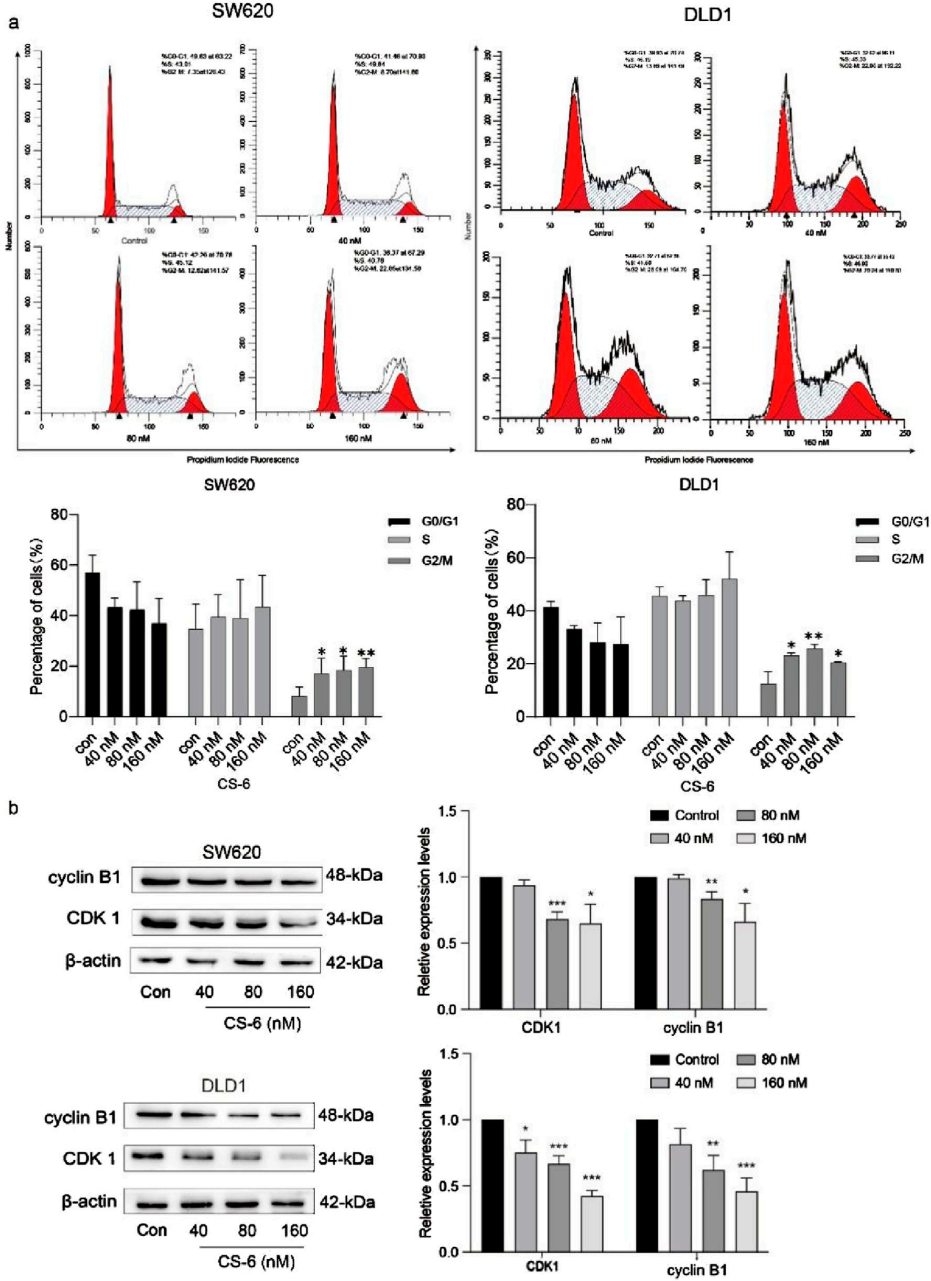
Illustration for the induction of G2/M phase arrest and alterations in the expression of cell cycle-related proteins upon exposure to CS-6 in SW620 and DLD1 cells. SW620 and DLD1 cells were exposed to different concentrations of CS-6 (40, 80, and 160 nM) for a 24 h, while the untreated cells were used as the Control group. **(a)** The representative histogram of the cell cycle distribution in the Control and CS-6-treated SW620 and DLD1 cells analyzed using flow cytometry. **(b)** Western blot analysis reveals the levels of cyclin B1 and cyclin-dependent kinase 1 in both the Control and CS-6-treated SW620 and DLD1 cells. The presented values indicate the mean ± standard deviation obtained from three independent experiments. Statistical significance is established at *p < 0.05, **p < 0.01, and ***p < 0.001.

To unravel the mechanism underlying CS-6-related inhibitory impact on CRC cell proliferation, PI staining was employed to scrutinize the distribution of cell cycle phases in CS-6-treated SW620 and DLD1 cells. The results revealed a significant induction of G2/M phase arrest in both SW620 and DLD1 cells when compared to the control treatment, as illustrated in Figure 2a.

The formation of the CDK1/cyclin B1 complex assumes a pivotal role in initiating the cyclic phosphorylation of various target substrates (Yin et al., 2017). This process is indispensable for advancing through the cell cycle. Therefore, WB assays were conducted to delve into the expression of cell cycle-related proteins, aiming to shed light on the mechanism behind CS-6-induced cell cycle arrest in CRC cells, as visually presented in Figure 2b.

The results indicated that CS-6 treatment led to a reduction in the expression levels of cyclin B1 and CDK1 relative to the control treatment in SW620 and DLD1 cells. In summary, these findings suggest that CS-6 exerts its inhibitory effect on CRC cell proliferation by promoting G2/M phase arrest.

### 3.3 CS-6 induced DNA damage in SW620 and DLD1 cells

**FIGURE 3:**
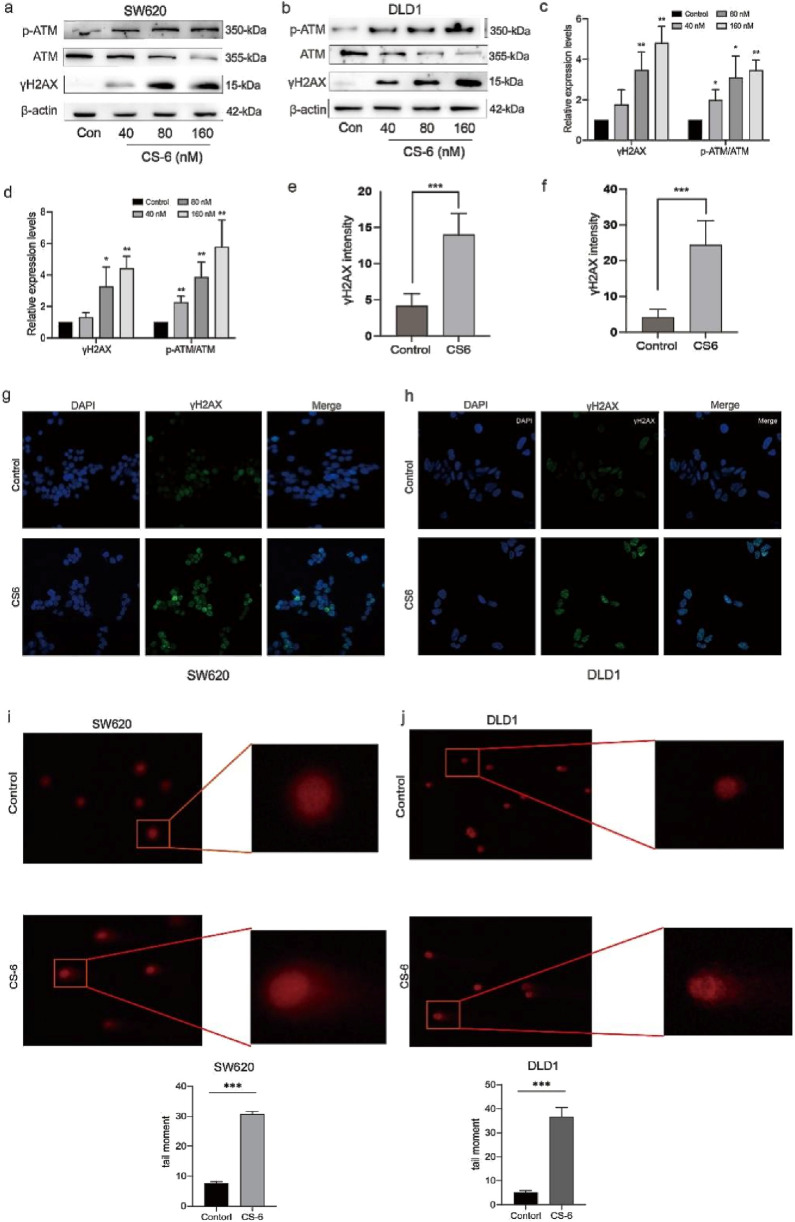
CS-6 induced DNA damage in SW620 and DLD1 cells. SW620 and DLD1 cells were treated with 80 nM CS-6 for 24 h. The untreated cells were used as Control group. **(a–d)** Western blot analysis was conducted to assess the levels of phosphorylated-ataxia telangiectasia mutated kinase (p-ATM), ATM, and γH2AX levels in both Control and CS-6-treated SW620 and DLD1 cells. **(e–h)** Confocal microscopy images were captured to visualize γH2AX and nuclear staining in Control and CS-6-treated SW620 and DLD1 cells, presented as single-channel and merged images. Scale bar = 5 µm. **(i,j)** The Comet assay was performed to illustrate CS-6-induced DNA damage in CRC cells. The presented values indicate the mean ± standard deviation obtained from three independent experiments. Statistical significance is established at *p < 0.05, **p < 0.01, and ***p < 0.001.

To determine whether CS-6-mediated cell cycle arrest in CRC cells is due to DNA damage, the expression of DNA damage markers in CS-6-treated SW620 and DLD1 cells was examined. Phosphorylation is a significant post-translational modification of proteins, and DNA double-strand breaks (DSBs) activate ATM kinase (Chen et al., 2022). The WB assay results revealed that the expression levels of DNA damage markers, γH2AX and p-ATM, were significantly upregulated after CS-6 treatment in both SW620 and DLD1 cells (Figure 3a-d). Furthermore, the IF assay showed increased fluorescence of FITC-labelled γH2AX foci after CS-6 treatment (80 nM), consistent with the WB results (Figure 3e-h). The Comet assay further confirmed that CS-6 induced DNA damage in both SW620 and DLD1 cells (Figure 3i, j).

### 3.4 CS-6 upregulated autophagy substrate p62 in SW620 and DLD1 cells

**FIGURE 4:**
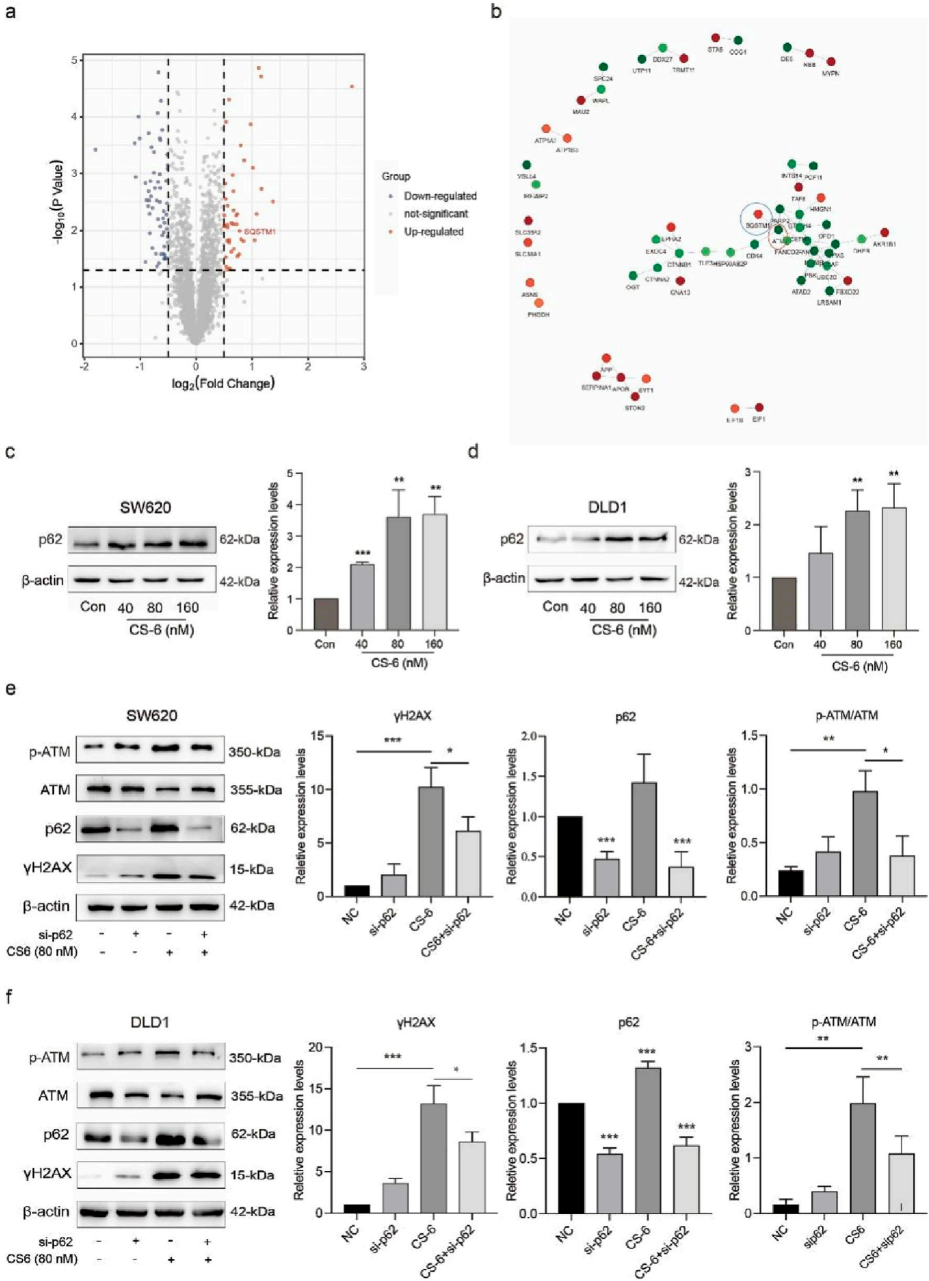
CS-6 upregulated p62 expression in SW620 and DLD1 cells. SW620 and DLD1 cells were treated with different concentrations of CS-6 for 24 h. The untreated cells were used as Control group. Additionally, SW620 and DLD1 cells were transfected with scrambled siRNA (Control) and p62 siRNA for 36 h, followed by treatment with 80 nM CS-6 for 24 h **(a,b)** Volcano map displaying differentially expressed genes and protein interactions in SW620 cells, assessed by proteomics analysis. **(c,d)** Western blot (WB) assay showing p62 levels in Control and CS-6-treated SW620 and DLD1 cells. **(e,f)** WB assay of DNA damage-associated proteins in Control and si-p62-transfected/CS-6-treated CRC cells. The presented values are the mean ± standard deviation obtained from three independent experiments. Significance levels are indicated as *p < 0.05 and **p < 0.01.

To further investigate the specific mechanisms by which CS-6 mediates DNA damage in CRC cells, proteomic analysis was conducted to identify differential proteins with CS-6. Studies have revealed that p62 is shuttled into the nucleus to mediate DNA damage and participate in DNA damage repair (Waterman et al., 2020). Proteomic analysis results indicated that Autophagy substrate-p62 was significantly upregulated in CS-6-treated SW620 cells (Figure 4a). Additionally, protein-protein interaction (PPI) analysis revealed a significant interaction between ATM and p62 (Figure.4b), while WB assays demonstrated the upregulation of p62 in both SW620 and DLD1 cells after CS-6 treatment (Figure 4c-d). To investigate the role of p62 in CS-6-treated CRC cells, transient transfection of SW620 and DLD1 cells with siRNA (si-p62) was performed to silence p62. WB analysis revealed that p62 knockdown increased cyclin expression and reduced the levels of DNA damage-associated proteins, including p-ATM and γH2AX, in CS-6-treated CRC cells (Figure 4 e-h). And after our test, non-silencing and sip62 did not have obvious growth inhibition on cells.(Fig. S1) In summary, these findings suggest that CS-6 may promote DNA damage in SW620 and DLD1 cells via the p62-regulated ATM/γH2AX pathway.

### 3.5 CS-6 induced autophagy and caused autophagic defect in SW620 and DLD1 cells

**FIGURE 5:**
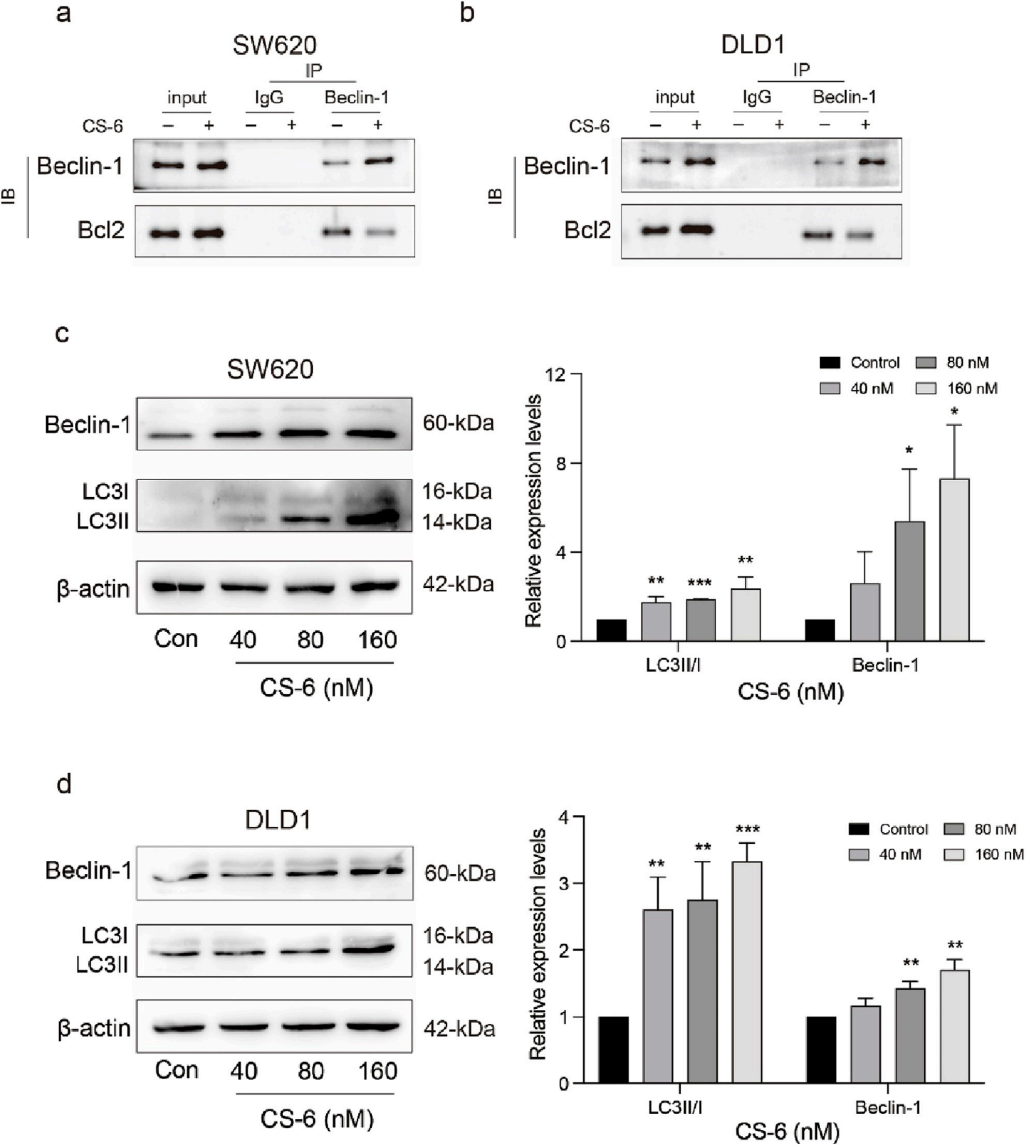
CS-6 induced autophagy and casused autophagic defect in SW620 and DLD1 cells. SW620 and DLD1 cells were treated with different concentrations of CS-6 for 24 h. Untreated cells served as the Control group. **(a,b)** Co-immunoprecipitation analysis was conducted to investigate the interaction between Beclin-1 (BECN1) and BcL2 in both Control cells and those treated with 80 nM CS-6. **(c,d)** Western blot analysis was performed to determine the levels of autophagy-related proteins (BECN1 and microtubule-associated proteins 1A/1B light chain 3B) in Control cells and those exposed to 40, 80, and 160 nM CS-6. The presented values indicate the mean ± standard deviation obtained from three independent experiments. Statistical significance is established at *p < 0.05, **p < 0.01, and ***p < 0.001.

In addition to its role in DNA damage and repair, p62 is also a known substrate for autophagy. Previous studies have demonstrated that upregulation of p62 may be associated with impairment of the downstream autophagic flux pathway and inhibition of autophagosome-lysosome fusion (Hewitt et al., 2016). Therefore, the role of CS-6 in activating autophagy in CRC cells was explored. Autophagy activation requires the release of BECN1 from the BECN1/BcL2 complex (Komatsu and Ichimura, 2010). The Co-IP assay showed that CS-6 disrupted the BECN1/BcL2 interaction in SW620 and DLD1 cells (Figure 5a,b). Additionally, the WB assay revealed that the expression of BECN1 and LC3-B significantly increased in SW620 and DLD1 cells after CS-6 treatment (Figure 5c,d) In summary, these findings suggest that CS-6 induced autophagy but impaired autophagic flux in SW620 and DLD1 cells.

### 3.6 CS-6-induced autophagy defects exacerbated DNA damage in SW620 and DLD1 cells

**FIGURE 6:**
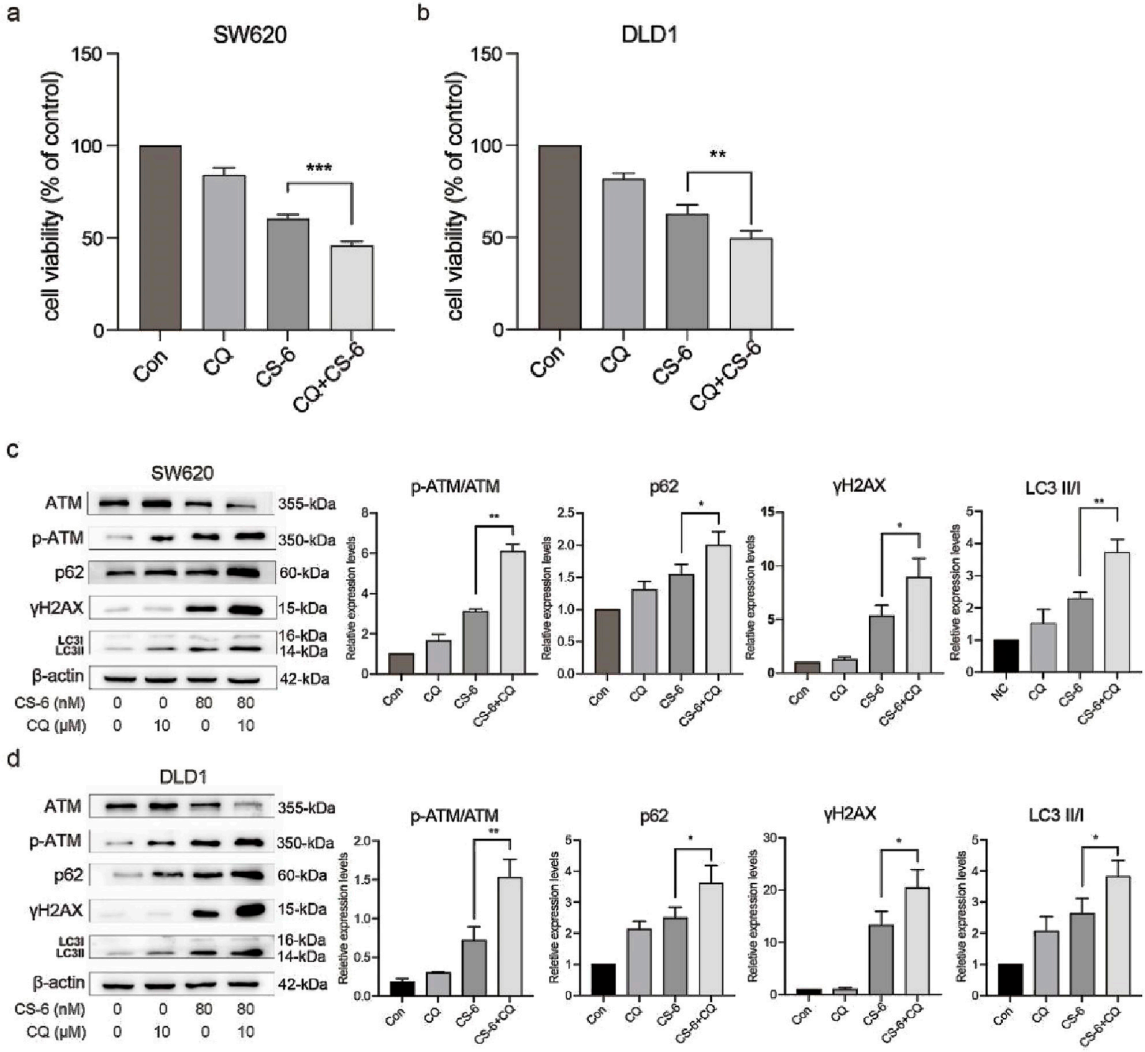
CS-6-induced autophagy defects exacerbated DNA damage in SW620 and DLD1 cells. SW620 and DLD1 cells were treated with 80 nM CS-6 and/or 10 μM chloroquine (CQ) for 24 h **(a,b)** Cell viability of CS-6-treated and CS-6/CQ-treated SW620 and DLD1 cells was measured using the CCK8 assay. **(c,d)** Western blot assay was conducted to measure the levels of phosphorylated-ataxia telangiectasia mutated kinase (p-ATM), ATM, and γH2AX in CS-6-treated and CS-6/CQ-treated SW620 and DLD1 cells. The presented values indicate the mean ± standard deviation obtained from three independent experiments. Significance levels are indicated as *p < 0.05 and **p < 0.01.

**FIGURE 7:**
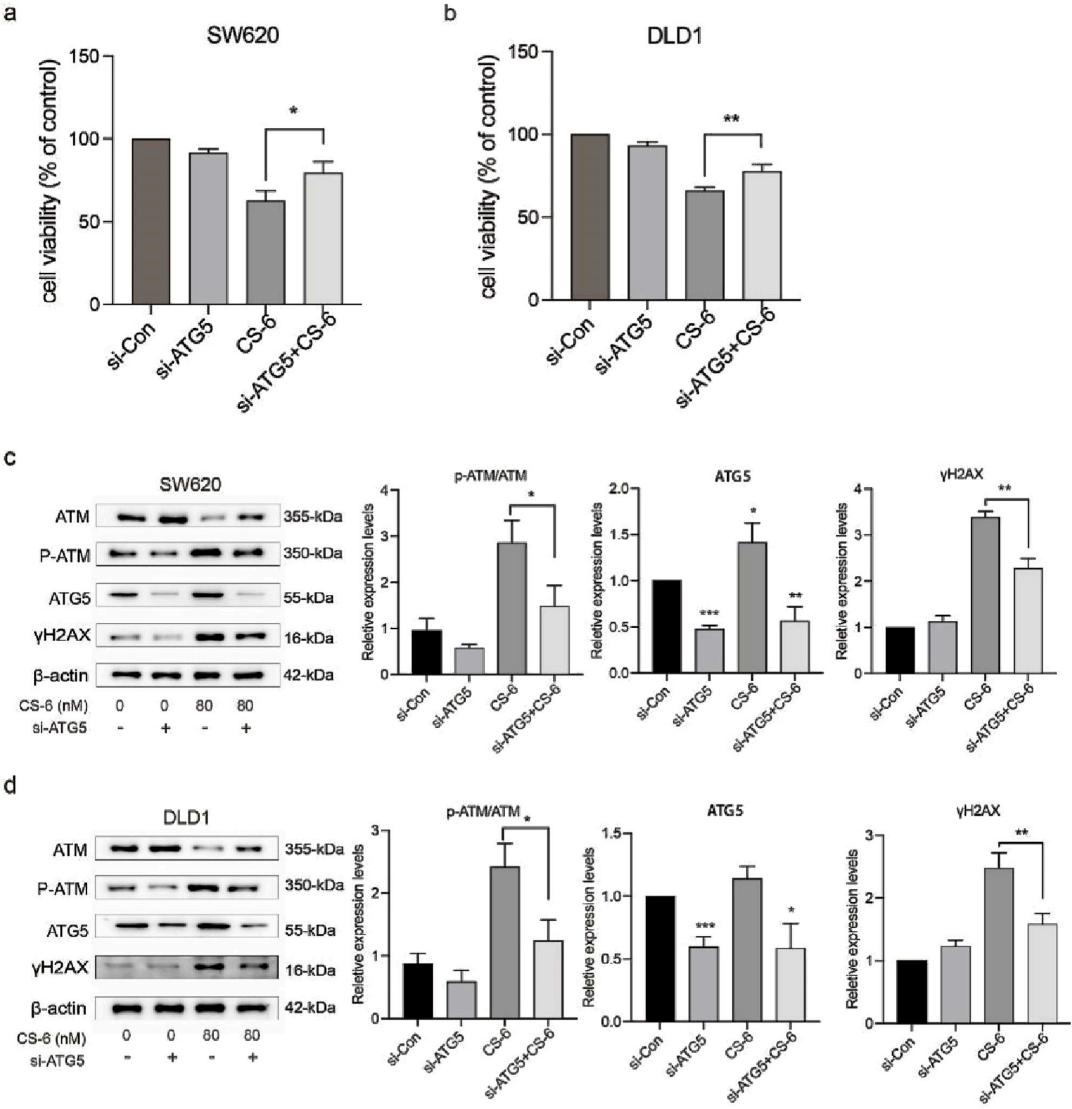
Inhibition of CS-6-induced autophagy reversed DNA damage in SW620 and DLD1 cells. SW620 and DLD1 cells were transfected with scrambled siRNA (Control) and si-ATG5 for 48 h, followed by treatment with 80 nM CS-6 for 24 h **(a,b)** Cell viability of Control and si-ATG5-transfected/CS-6-treated SW620 and DLD1 cells was measured using the CCK8 assay. **(c,d)** Western blot assay was conducted to measure the levels of phosphorylated-ataxia telangiectasia mutated kinase (p-ATM), ATM, and γH2AX in Control and si-ATG5-transfected/CS-6-treated SW620 and DLD1 cells. The values presented indicate the mean ± standard deviation obtained from three independent experiments. Significance levels are indicated as *p < 0.05, **p < 0.01, and ***p < 0.001.

To further explore the crosstalk between CS-6-induced DNA damage and autophagy in CRC cells, SW620 and DLD1 cells were treated with CQ, a late-autophagy flux inhibitor. The results revealed that combined treatment with 10 μM CQ and 80 nM CS-6 significantly reduced the cell viability of CRC cells compared to CS-6 treatment alone (Figure 6 a,b). Moreover, combined CS-6/CQ treatment significantly increased LC3I/II and p62 levels in CRC cells, indicating a synergistic effect on the inhibition of autophagic flux (Figure 6 c,d). Furthermore, CQ treatment aggravated the CS-6-induced DNA damage in CRC cells (Figure 6 c,d). ATG5 is an autophagy-related protein required for the formation of autophagosomes (Fernández et al., 2018). Transient transfection of ATG5 siRNA (si-ATG5) to inhibit autophagic activity not only restored the cell viability of SW620 and DLD1 cells but further attenuated the CS-6-induced DNA damage in CRC cells (Figure 7a–d). Overall, these results indicate that CS-6-mediated autophagy aggravates DNA damage in CRC cells, and suppression of autophagic activity could enhance the anti-CRC effect of CS-6.

### 3.7 CS-6-mediated colorectal cancer suppression *in vivo* is associated with DNA damage and autophagy

**FIGURE 8:**
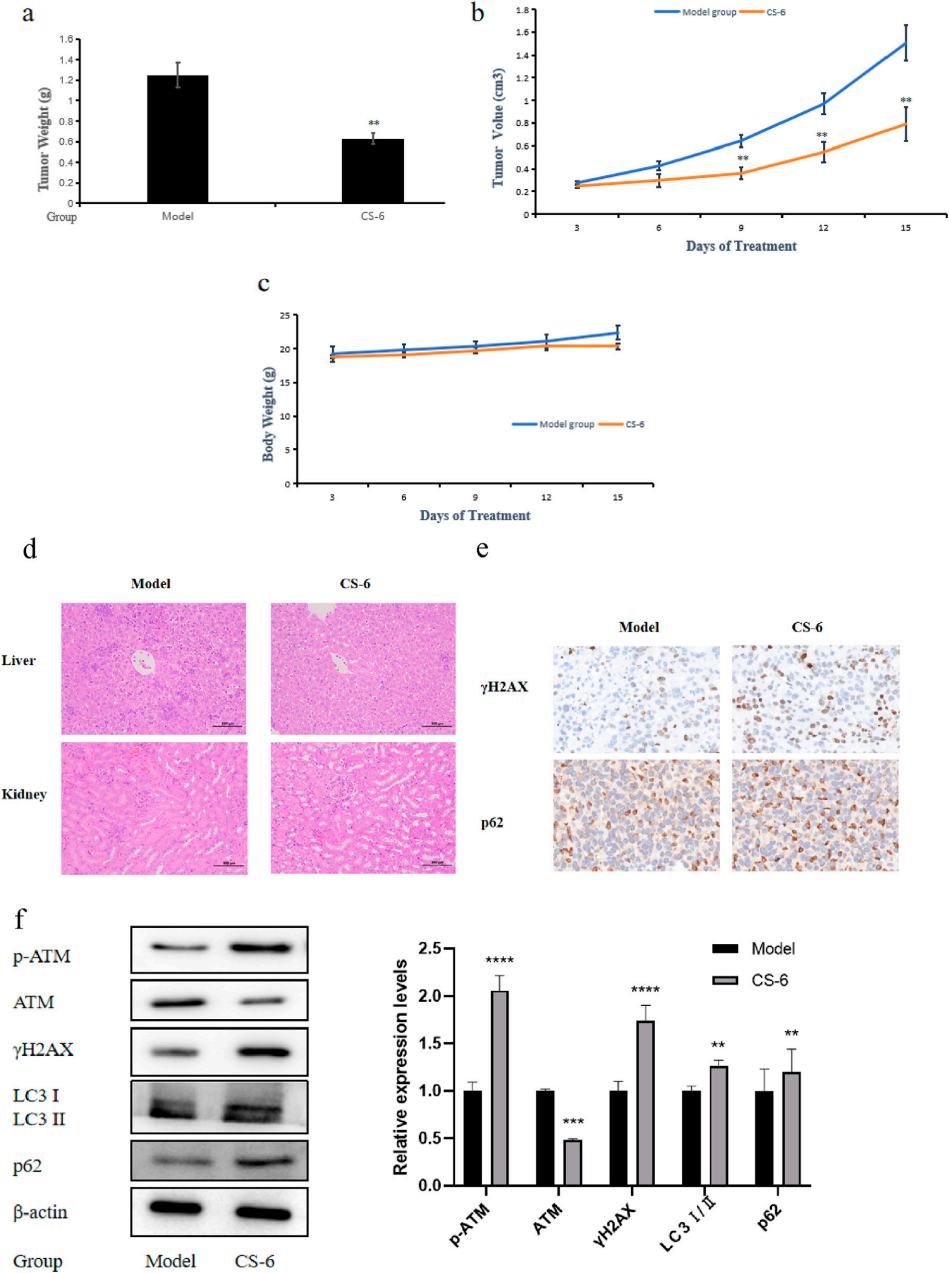
CS-6-mediated colorectal cancer suppression in vivo is associated with DNA damage and autophagy. **(a)** Tumor weight of SW620 cells in vivo. **(b)** Tumor volume of SW620 cells in vivo. **(c)** Body weight change of BALB/C nude mice. **(d)** Histological changes of liver and kidney in BALB/C nude mice treated with CS-6. **(e)** Expression of γH2AX and p62 was determined by immunohistochemistry (IHC). Data are presented as the mean ± SD from three independent experiments. **(f)** Expression of p-ATM, ATM, γH2AX, LC3 I/II and p62 in xenograft tumor at protein levels was detected by western blotting. *P < 0.05;; **P < 0.01; ***P < 0.001 compared with model group.

Firstly, we established a SW620 subcutaneous transplant tumor model in BALB/c-Nude mice and injected CS-6 intraperitoneally for 15 consecutive days after tumor formation. Figure 8a, b illustrate that CS-6 can significantly inhibit the growth of colorectal cancer in nude mice. Meanwhile, the results of tumor volume changes indicate a significant statistical difference starting from the ninth day of administration. Considering whether the administered dose of CS-6 was toxic to mice *in vivo*, we also evaluated its effect on the body weight and internal organs of mice. We found that CS-6 treatment had no effect on the body weight of the mice (Figure 8c). In particular, the relevant tests on the vital organs (liver and kidney) of the mice also showed no visible difference between the model and the administered groups, indicating that treatment with CS-6 at this dosage is nontoxic (Figure 8d). Concurrently, indicators related to DNA damage and autophagy in mice after administration of CS-6 were further measured. Consistent with the results of *in vitro* experiments, IHC analysis showed dramatic upregulation of γH2AX and p62 in the administered group compared to the model group (Figure 8e). Consistent with *in vitro* experiments, intraperitoneal injection with CS-6 promoted the protein expression of γH2AX and p62 by western blotting. Meanwhile, CS-6 promoted the protein expression of p-ATM and LC3 I/II. This implies that administration of CS-6 inhibited the growth of colorectal cancer *in vivo* by inducing autophagy defects, resulting in p62 accumulation and DNA damage.

## 4 Discussion

The occurrence of DNA damage stems from various factors, such as disruptions in cellular metabolism, exposure to toxins, or exposure to ultraviolet radiation. When DNA damage remains unaddressed, it can trigger programmed cell death mechanisms like apoptosis, autophagy, and necrosis. The role of autophagy in DNA damage includes the following aspects: autophagy reduces the spread of DNA damage by encapsulating and degrading damaged DNA fragments or abnormal proteins. This mechanism has been proven to maintain genomic stability and promote cancer cell survival in the treatment of colorectal cancer. In addition, autophagosomes can isolate damaged mitochondria and reduce DNA damage caused by mitochondrial dysfunction (Hewitt et al., 2016). In addition, autophagy enhances the cell’s ability to repair DNA damage by regulating the expression and activity of DNA repair enzymes such as DNA polymerase. Autophagy degradation products (such as amino acids and nucleotides) can provide essential materials for DNA repair and support the repair process by releasing energy (Fernández et al., 2018; Nishida et al., 2009). Thus, the function of DDR pathway is a critical defense mechanism, identifying DNA damage and initializing DNA repair processes to preserve genomic integrity.

As can be seen from Figure 8, we found that CS-6 could inhibit the tumor volume of colorectal carcinoma in nude mice without significant toxic effects, and the protein expression levels of γH2AX and p62 were also elevated *in vivo* after CS-6 treatment. To elucidate the mechanisms underlying the effects of CS-6 in CRC cells, a proteomics analysis was executed, suggesting a significant increase in p62 levels in CS-6-treated CRC cells. p62 is a stress-responsive protein that modulates the autophagic degradation process and nucleocytoplasmic shuttling. During the process of autophagy, p62 is selectively engulfed into autophagosomes and subsequently degraded by proteolytic enzymes, resulting in the reduced level of p62. Therefore, p62 level are inversely correlated with autophagic activity. Previous study found that nuclear targeting of p62 is required for increased DNA damage and growth inhibition mediated by RNF4 silencing (Nishida et al., 2009). Our findings indicated that CS-6 could activate autophagy in CRC cells by disrupting the BECN1/BcL2 complex. However, the accumulation of p62 in CRC cells suggested that CS-6 treatment disrupted the autophagic flux in CRC cells (Figure 6). Studies have shown that p62 has been linked to DNA DSB repair. Defects in autophagy following DNA damage are associated with histone H2A ubiquitination. The cytoplasmic accumulation of p62 in autophagy-deficient cells impedes the recruitment of DNA repair proteins, including BRCA1, RAP 80, and Rad 51, to the DNA DSB binding sites, thus hindering DNA repair and exacerbating DNA damage (Lv et al., 2022). Our results demonstrated that the combined treatment of CS-6 with CQ not only synergistically inhibited autophagic flux but also intensified DNA damage in CRC cells. Furthermore, the knockdown of ATG5, an autophagy-related protein, reduced DNA damage in CRC cells. These outcomes suggest that CQ amplifies DNA damage by promoting p62 accumulation, blocking late-stage autophagy exacerbates CS-6-induced DNA damage, and blocking autophagy suppresses CS-6-induced DNA damage. Consequently, CS-6-induced p62 accumulation can lead to severe DNA damage that remains unrepaired, further escalating DNA damage.

**FIGURE 9:**
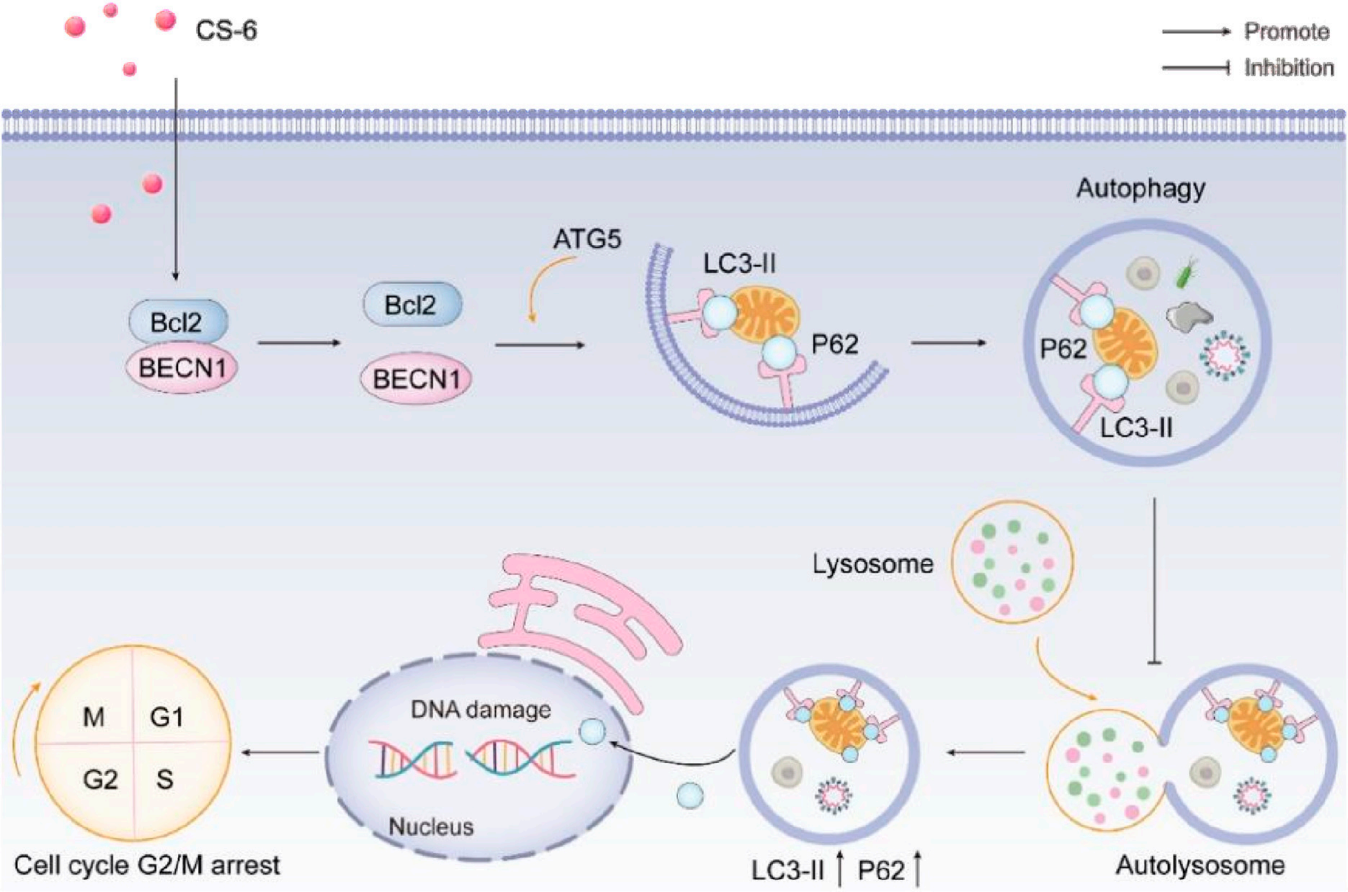
Schematic representation of the anti-tumor effect of CS-6 in colorectal cancer.

CS-6, the primary steroidal compound found in Chansu, exhibits substantial anti-tumor activity with minimal toxicity and side effects. It inhibits cell proliferation, induces apoptosis, and regulates immune responses in various cancer types (Komatsu et al., 2007). Previous research has unveiled several roles of CS-6 in different cancers, including its inhibits IKKβ phosphorylation by targeting ATP binding sites, thereby inhibiting NF-κB binding and p300 recruitment of COX-2 promoters, thereby strongly inhibiting COX-2 expression in lung cancer (Lan et al., 2020). Its potential in glioblastoma treatment in combination with other drugs (Yu et al., 2014). CS-6 could inhibited osteosarcoma cells viability and tumorigenesis capability by blocking the TGF-βPI3K/AKT signaling pathway (Yuan et al., 2021; Ma et al., 2020), and its capacity to degrade c-Myc in multiple myeloma cells (Liu et al., 2017). However, studies investigating the underlying anti-cancer effects and mechanisms of CS-6 in CRC have been lacking. Our study demonstrates that CS-6 exerts a potent anti-tumor effect on SW620 and DLD1 cells by inducing DNA damage, causing autophagy defects, promoting p62 accumulation, and impeding the repair of DNA, thereby exacerbating DNA damage (Figure 9). In conclusion, our findings suggest that CS-6 holds significant anti-cancer potential in treating CRC and may serve as a promising therapeutic agent for managing this condition.

## Data Availability

The datasets generated during and/or analyzed during the current study are available from the corresponding author on reasonable request.
